# The utility of ctDNA in rectal cancer

**DOI:** 10.1093/oncolo/oyaf354

**Published:** 2025-10-27

**Authors:** Gagandeep Brar, Pashtoon Murtaza Kasi

**Affiliations:** Department of Medical Oncology & Therapeutics Research, City of Hope Comprehensive Cancer Center, Duarte, CA, United States; Department of Medical Oncology & Therapeutics Research, City of Hope Comprehensive Cancer Center, Duarte, CA, United States

**Keywords:** ctDNA, rectal cancer, total neoadjuvant therapy, complete response, metastases

The management of locally advanced rectal cancer (LARC) has changed dramatically over the last several years. In what used to be a “one size fits all” approach using chemoradiation followed by surgery followed by adjuvant chemotherapy in all comers with newly diagnosed rectal adenocarcinoma, the field has now converted to eligible patients receiving up front total neoadjuvant therapy (TNT) followed by surgery or, in good responders, a non-operative organ preserving watch and wait approach.^1^ The goal of TNT is to improve surgical outcomes, decrease local recurrence and distant metastases. Factors that influence decision-making for TNT include tumor stage, location, imaging, endoscopic and pathologic response as well as patient preference. However, the local recurrence rate remains around 4%-8% and distant metastases 20%-25%, likely due to variations in patient selection, definitions of staging and response, ability to complete treatment and institutional practices.^2–5^

Circulating tumor DNA (ctDNA) is an emerging biomarker for recurrence in stage II and III colon cancers offering potential advantages in diagnosis, prognosis and treatment monitoring.^6–10^ Potential usages of ctDNA include monitoring for disease progression or early recurrence and treatment decision-making (e.g. escalation or de-escalation). Two methodologies for assessing ctDNA exist: tumor informed and tumor agnostic. In tumor informed testing, tumor tissue is sequenced to identify patient specific mutations for monitoring. This tends to result in higher sensitivity and specificity but at the cost of expense and time. In tumor agnostic testing, tissue is not required, and testing is performed using a broad panel of common mutations or alterations, which is usually less expensive and quicker to result. Tumor agnostic testing may allow for broader applicability, but its sensitivity is usually lower. In addition, false positives and negatives can occur, which can lead to under- or over-treatment of patients. In early-stage colon cancer, ctDNA has a strong association with prognosis and overall survival. However, in rectal cancer, the data is more limited and has been proposed as a tool for detecting minimal residual disease (MRD), guiding treatment decisions and predicting recurrence.^11–13^ Looking at the recent landscape, we want to highlight a few pivotal studies in this space and review the challenges of utilizing ctDNA in patients with rectal cancer.

The AGITG DYNAMIC-Rectal study was a multi-center randomized controlled phase 2 study where patients with cT3-4 and/or lymph node positive disease were treated with chemoradiation followed by surgery.^14^ In patients who were eligible for adjuvant chemotherapy, a tumor informed ctDNA guided approach was utilized, specifically, patients who tested ctDNA positive were treated with adjuvant chemotherapy per standard of care. For ctDNA negative patients, patients were able to forego chemotherapy if lymph node status was down staged to N0 post operatively. If they remained lymph node positive, then it was the choice of the treating physician whether to proceed with adjuvant chemotherapy. The study was small and terminated early due to a paradigm shift towards total neoadjuvant therapy, however, it did suggest that a ctDNA guided approach for adjuvant chemotherapy may be feasible. In addition, the study exhibited an innovative way of pairing decisions in patients with negative ctDNA results with pathologic response achieved post chemoradiation. In higher risk pathology, as ctDNA can be falsely negative, adjuvant chemotherapy was still recommended.

In a study by Zhou and colleagues, patients with locally advanced rectal cancer were evaluated with tumor informed ctDNA after neoadjuvant chemoradiation.^13^ Although small numbers, patients who achieved a pathologic complete response had no pre-operative ctDNA that could be detected. The authors also found that patients who never cleared their ctDNA were associated with a shorter metastasis- free survival, suggesting a correlation between ctDNA and minimal residual disease. The GEMCAD 1402 was a similar study evaluating pre-surgery tissue agnostic ctDNA in patients with LARC.^11^ However, there was no association found between ctDNA and pathologic response to therapy, possibly related to low sensitivity (0.75). However, detectable persistent ctDNA did correlate to a higher rate of recurrence and shorter survival. Lastly, a study by Murahashi and colleagues also showed persistent ctDNA positivity was an interpedently prognostic marker for recurrence post operatively.^12^

The NEO (CCTG CO.28) study was recently presented and is of particular interest.^15^ Patients with cT1-3 node negative rectal cancers were treated with neoadjuvant chemotherapy then evaluated by tumor agnostic ctDNA with the goal of organ preservation. Twenty-eight patients were enrolled and ctDNA was detected in 46% of samples. In patients who cleared their ctDNA, most underwent transanal excision surgery. In all five recurrences, ctDNA was difficult to detect or was negative prior to neoadjuvant chemotherapy, suggesting limited sensitivity.

A recent study published by Alden and colleagues looked to evaluate tumor informed ctDNA as a predictor of pathologic, radiographic and endoscopic response after TNT for patients that could then be candidates for non-operative management.^16^ This was a single center, retrospective study evaluating 44 patients with LARC defined as cT3-4NxM0 or cTxN1-2M0 rectal cancer treated with total neoadjuvant therapy including short-course radiotherapy (SCRT) followed by doublet chemotherapy or long-course radiotherapy (LCRT) followed by doublet chemotherapy. Response was defined by post treatment MRI tumor regression grade (mrTRG) and endoscopy and ctDNA samples were collected at multiple time points. Twenty-two patients were evaluable with pre-treatment ctDNA. Levels were detectable in 18 (82%) patients and post treatment ctDNA remained detectable in 3 (13%) patients. The authors found that post-TNT ctDNA had a sensitivity of 23% and specificity of 100% for predicting residual disease after resection (compared to MRI with sensitivity of 62% and specificity of 22% and endoscopy with sensitivity of 58% and specificity of 11%), post-TNT ctDNA had a sensitivity of 16% and specificity of 96% in predicting poor tumor regression based on mrTRG, post-TNT ctDNA had a sensitivity of 5% and specificity of 95% in predicting residual disease based on endoscopic response, and all patients with complete/good response on imaging had undetectable post-TNT ctDNA. The authors concluded that in real world practice, ctDNA had poor sensitivity and negative predictive value in detecting residual disease after TNT, suggesting that ctDNA alone is insufficient to predict which patient can forego surgery and be monitored with non-operative management.

In another study by Fakih et al., patients with resected colorectal cancers were analyzed who underwent surveillance by imaging and CEA per standard practice guidelines as well as ctDNA.^17^ Forty-eight patients were followed using the Signatera ctDNA assay. The authors found that the sensitivity of imaging and CEA was better than ctDNA in identifying disease recurrence (73.3% vs 53.5%). There was also no statistical difference in identifying a true recurrence across imaging, CEA and ctDNA.

**Figure 1.**
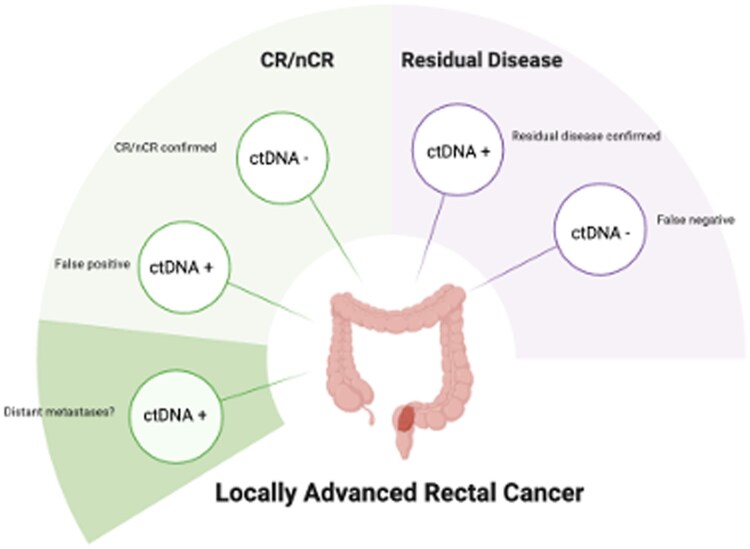
Possible clinical scenarios based on ctDNA results. cCR: clinical complete response; nCR: near clinical complete response.

There may be many reasons as to why ctDNA fails to detect cancer including limitations in sensitivity as mentioned above, lack of standardization and methodologies of ctDNA assays (tissue informed vs tissue agnostic), as well as cost and accessibility concerns. As we learn more about utilizing ctDNA in a real-world practice, we’d like to highlight some possible scenarios of using ctDNA in patients with locally advanced rectal cancer (Figure 1).

1. ctDNA negative and complete response (CR)/near complete response confirmed (nCR): This is the ideal scenario where ctDNA correctly correlates with patients who achieve a complete or near complete clinical response, suggesting high sensitivity. These patients would be the ideal candidates to discuss a watch and wait approach where surgery can be safely omitted with close surveillance
2. ctDNA positive and CR/nCR confirmed: This would be an example of a false positive.
3. ctDNA positive and CR/nCR confirmed: This would be a patient where distant metastases would need to be ruled out. If patient remains ctDNA positive, then this may be a false positive or is an indicator of minimal residual disease with high risk of recurrence/distant disease in the future. This may be a scenario where a more aggressive surveillance program should be considered.
4. ctDNA positive and residual disease confirmed: This would be another scenario where ctDNA positivity correctly correlates with residual disease being present, suggesting high specificity. In this context, patients would be recommended to proceed with surgical resection.
5. ctDNA negative and residual disease confirmed: This would be an example of a false negative.

There are two prospective studies currently ongoing that may better answer the question as to whether ctDNA has a predictive role in LARC management (GEMCAD-REVEAL and JANUS). It would be interesting to follow these patients post-neoadjuvant treatment to see if ctDNA can correlate with locoregional recurrence/failure or possibly distant metastases. Table 1 summarizes additional ongoing studies evaluating ctDNA in LARC.

**Table 1.** Ongoing studies evaluating ctDNA in rectal cancer.

NCT	Title	Assay type	Patient number	Overview
**NCT06589388**	ctDNA Monitoring to Predict the Efficacy of TNT for Rectal Cancer	ctDNA	100	Primary outcome: disease-free survival
**NCT05487248**	A Study of On-treatment CtDNA Changes in Chemo-refractory Colorectal Cancer Patients (COPERNIC)	ctDNA (FoundationOne)	109	Correlating ctDNA with treatment outcomes and survival Combination of colon and rectal cancer patients
**NCT05629442**	ctDNA and Organ Preservation/​Pathologic CR in Rectal Cancer	ctDNA	—	Evaluating for minimal residual disease
**NCT02579278**	Circulating Tumour DNA (ctDNA) in Patients With Colorectal Cancer and the Relationship to Imaging Features of Extramural Venous Invasion (ctDNA)	ctDNA	100	Evaluating correlation between ctDNA and vascular spread
**NCT05601505**	Circulating Tumor DNA-guided Neoadjuvant Treatment Strategy for Locally Advanced Rectal Cancer (CINTS-R)	ctDNA	470	Therapeutic intervention based on ctDNA (neoadjuvant chemoradiation vs TNT)
**NCT05081024**	Establishing a ctDNA Biomarker to Improve Organ Preserving Strategies in Patients With Rectal Cancer	ctDNA (Natera)	50	Primary objective: cCR
**NCT03749083**	A Study of the Role of Circulating Tumor DNA in Predicting the Likelihood of Organ Preservation After Clinical Complete Response to Neoadjuvant Therapy for Rectal Cancer	ctDNA	60	Evaluating correlation between ctDNA and organ preservation
**NCT05674422**	GEMCAD-REVEAL STUDY - Circulating Tumor DNA as a Predictor of Relapse in Patients With Locally Advanced Rectal Cancer (REVEAL)	ctDNA	120	Evaluating ctDNA and relapse in patients who undergo TNT followed by WW or TME
**NCT05610163**	Testing the Addition of an Anti-Cancer Drug, Irinotecan, to the Standard Chemotherapy Treatment (FOLFOX) After Long-Course Radiation Therapy for Advanced-Stage Rectal Cancers to Improve the Rate of Complete Response and Long-Term Rates of Organ Preservation (JANUS)	ctDNA	312	Primary objective: cCR

In conclusion, the utility of ctDNA in locally advanced rectal cancer remains under investigation. Herein we summarized the data and specific considerations in patients with non-metastatic rectal cancer. Key observations as cited by many authors of some of these publications is to use ctDNA as an adjunct to standard testing strategies. In the context of patients with ctDNA positive disease, at present, assessing where ctDNA shedding is coming from (e.g. primary site vs occult metastases) is not provided at the commercial level. However, due to specific epigenomic or methylation signatures, this may be feasible and hence be more of an actionable marker to address where to look for residual or metastatic cancer.
